# Materia Medica and Therapeutics

**Published:** 1879-09

**Authors:** 


MATERIA MEDICA AND THERAPEUTICS—VEGETABLE KINGDOM. By Chas. D. F. Phillips, M.D., F.R.C.S.E., Lecturer on Materia Medica, Westminster Hospital, London. Edited and adapted to the U. S. Pharmacopœia. By Henry G. Piffard, M.D., Prof. of Dermatology Univ. of N. Y., etc. Wm. Wood & Co., N. Y. 1879.

The series of the "Library of Standard Authors" could not be complete without some work on materia medica. In the work before us, there is evidence of its adaptability, as found in the compendious arrangement and comprehensive disposition of the subject matter. The classification is founded more on botanical considerations, than on medical, yet the physiological and therapeutical action of each plant is given in detail. Supplemented to each individual description, is a list of the medicinal preparations into which the article enters, and the dose of each. The work is devoted exclusively to the consideration of vegetable agents, and with few exceptions, contains all the known medical plants.

